# Changes of apoptosis regulation in the endometrium of infertile women with tubal factor and endometriosis undergoing in vitro fertilization treatment

**DOI:** 10.5935/1518-0557.20140084

**Published:** 2014

**Authors:** Yulia S. Antsiferova, Natalia Yu. Sotnikova, Irina K. Bogatova, Alisa V. Boitsova

**Affiliations:** 1 Research Institute of Maternity and Childhood - Ivanovo, Russian Federation

**Keywords:** IVF, endometrium, apoptosis, tubal factor, endometriosis

## Abstract

**Objective:**

To establish the relationship between the endometrial apoptosis parameters and endometrial receptivity in infertile women undergoing IVF treatment.

**Methods:**

73 women with tubal infertility, 27 infertile women with endometriosis and 13 healthy fertile women (control group) were recruited into the study. 53 women with tubal factor of infertility and 17 patients with endometriosis later entered in the IVF protocol. Samples of endometrial tissue were used as the material for investigation. Endometrium was collected on the 20-24 days of non-conceptual cycle, using the Pipell suction curette. XIAP, PTEN and HSP27 mRNAs expression in the endometrial tissue was assessed by real time RT-PCR.

**Results:**

In women with tubal infertility the high level of XIAP, HSP27 and PTEN mRNAs expression in the endometrium was found. In infertile women with endometriosis the increase of XIAP and HSP27 mRNAs expression was noted. Success of the IVF outcome in women with tubal infertility was associated with maximal level of PTEN synthesis and in endometriosis group the pregnancy achievement after IVF treatment was noted in women with lower expression of XIAP mRNA.

**Conclusion:**

The high level of pro-apoptotic factors synthesis in the endometrium during window of implantation is associated with the readiness of endometrium to implantation.

## INTRODUCTION

Today in vitro fertilization (IVF) is widely used for the treatment of infertility in couples with unexplained subfertility, male subfertility, endometriosis or tubal pathology (Van Loendersloot *et al.*, 2010). But analysis of the data received from different clinical centers shows that the pregnancy rate after IVF treatment is still rather low and does not exceed 40-50% per embryo transfer cycle (Strowitzki *et al.*, 2006). The impact of some different clinical factors such as infertility etiology, female age, duration of infertility, number of 10-14-mm follicles, and progesterone level on the day of hCG injection on the outcome of the IVF treatment has been proven (Van Loendersloot *et al.*, 2010; Coccia *et al.*, 2011; Kummer *et al.*, 2011; Cai *et al.*, 2011). Endometrial receptivity also is estimated as the important factor of the IVF success, because to achieve a successful implantation, pregnancy and subsequent live child birth, endometrium should be able to accept the embryo (Strowitzki *et al.*, 2006; Garrido *et al.*, 2002). It is known that during menstrual cycle the human endometrium undergoes morphologic and biochemical modifications until a receptive endometrium is developed (Strowitzki *et al.*, 2006). Apoptosis helps to maintain the cellular homeostasis in the endometrium during the menstrual cycle. During the proliferative phase of menstrual cycle, which is characterized by the active cells proliferation and angiogenesis, apoptotic cells practically are absent in the endometrium (Garrido *et al.*, 2002). Supposedly the high level of Bcl-2 (B cell lymphoma/leukaemia-2) expression leads to the inhibition of apoptosis in the endometrium during this phase of cycle (Harada *et al.*, 2004). In the secretory phase of cycle, namely during the short period of endometrial receptivity, known as the “window of implantation”, the first signs of apoptosis in the glandular epithelia take place (Szmidt *et al.*, 2010).

In humans the endometrium becomes receptive to blastocyst implantation at 6-8 days after ovulation and remains receptive for approximately 4 days (cycle days 20-24) (Szmidt *et al.*, 2010).

Normally apoptosis of endometrial cells is significantly increased at this period, providing the successful invasion of blastocyst by locally induced cells death in the site of the embryo and endometrial surface contact (Rashid *et al.*, 2011). With progressing of implantation, the regression of decidual cells allows a restricted and coordinated invasion of trophoblast cells into the maternal compartment due to the balanced expression of Bax, Bcl-2 and caspase-9 proteins in the decidual compartment and the high level of caspase-3 synthesis in the apoptotic uterine epithelium (Joswig *et al.*, 2003). So, apoptosis regulation is crucial for the embryo implantation. But it is obvious that many apoptosis-regulating factors directly implicated in the cross-talk between the embryo and endometrium preceding implantation have not yet been elucidated.

The relationship between the endometrial apoptosis, readiness of endometrium to implantation and IVF outcome are not yet clear.

In our work we attempted to: a) define the level of the expression of mRNAs of factors with pro- and anti-apoptotic activity in the endometrium of women with tubal infertility and with endometriosis-associated infertility, b) compare the rate of IVF success in groups of women with tubal infertility and endometriosis-associated infertility; c) retrospectively analyze the character of apoptosis regulation in the endometrium of infertile women with different outcome of IVF treatment to elucidate the possible new endometrial markers of IVF success.

## MATERIALS AND METHODS

### Patients

Women undergoing IVF treatments at the Center of Family Planning of Ivanovo State Research Institute of Maternity and Childhood between 2009 and 2011 years were recruited into the study. The first study group consisted of 73 women with tubal factor infertility, aged 33.13±0.42 years. The second study group consisted of 27 women with infertility associated with endometriosis, aged 33.14±1.02. Diagnosis of endometriosis was earlier confirmed by laparoscopic investigation. 13 gynecologically healthy women with proved fertility, aged 30.60±1.66, who were admitted for tubal ligation, were taken into the investigation as the control group.

Samples of endometrial tissue were used as the material for investigation. Bioptats of endometrial tissue were received at the time of “window of implantation” (cycle days 20-24) of a non-conceptual cycle. On the same day of endometrial sampling serum levels of FSH and LH were determined. In infertile women samples of the endometrial tissue were taken 1-4 months before IVF treatment. Biopsy was performed using the Pipell (De Cornier, Laboratoire C.C.D., France) suction curette. All patients had regular menstrual cycle and were not taking any hormone therapy at the time of biopsy. The Ethics Committee of Ivanovo State Research Institute of Maternity and Childhood approved this study and each woman participating in our study gave informed consent.

53 women with tubal factor of infertility and 17 patients with endometriosis later entered in the IVF protocol. Ovarian hormonal stimulation was conducted according to a standard “long” protocol using gonadodrophin-releasing hormone (GnRH) agonist (Diphereline daily; Ipsen, France) and recombinant follicle-stimulating hormone (Gonal-F;Merck Serono Switzerland).

Serum hCG was checked 14 days after embryo transfer and patients with a hCG level (>50 IU/L) were considered pregnant. An ultrasound scan was performed one week later and then again three weeks later in order to determine the number of intrauterine gestational sacs present and fetal viability, respectively.

All the pregnant women subsequent to IVF treatment were followed until the termination of pregnancy or live birth. A living child one week after delivery is defined as a live birth.

### Real-time RT-PCR

Total RNA was isolated from the whole endometrial tissue using the standard guanidinum thiocyanate-phenol-chloroform method (Van Velden *et al.*, 2003). RNA was converted to complementary DNA (cDNA) using random hexamers and murine leukaemia virus reverse transcriptase (Promega, USA). For real time quantitative RT-PCR the commercial sets of gene-specific primers and probes for β2-microglobulin (housekeeper gene), X-linked inhibitor of apoptosis (XIAP), phosphatase and tensin homolog, deleted on chromosome 10 (PTEN) and heat shock protein 27 (HSP27) (Sintol, Moscow, Russia) were used.

For the thermocycle reactions and the detection of the fluorescence signals iCycler iQ Multi-Color Real Time PCR detection System (BIO-RAD Laboratories, California, USA) was used.

The amount of copy numbers of cDNAs of specific genes was assessed using the control cDNA dilution series. For each sample the amount of copy numbers of β2-microglobulin and specific genes were determined from the appropriate standard curve generated by iCycler iQ software. The amount of specific gene was subsequently divided by the housekeeper gene amount to obtain the normalized specific gene value and results were presented as the ratio in a sample x 10^1^ per µl for PTEN and as the ratio in a sample x 10^4^ per µl for HSP27 and XIAP.

### Statistics

Data were estimated using STATISTICA 6.0 software. Results for the level of mRNAs expression were presented as the mean ± standard error. Statistical analysis was performed using Student’s t-test for parametric variables and the chi-square test for categorical variables. A *P* value <0.05 was considered statistically significant.

## RESULTS

### Expression of pro- and anti-apoptotic factors mRNAs in the endometrium of infertile women with tubal factor and endometriosis

Our results evidence that in groups of infertile women with tubal factor and endometriosis the regulation of apoptosis was impaired (Table 1). In women with tubal factor of infertility the increased levels of the expression of mRNAs of pro-apoptotic factor PTEN and also of anti-apoptotic factors XIAP and HSP27 were noted comparing to that in healthy women (*P*<0.05 in all cases). In the group of infertile women with endometriosis the significantly higher level of the XIAP and HSP27 mRNA expression was found comparing to that in the control group (*P*<0.05 in both cases). The comparative analysis of the endometrial gene profile expression in two groups of infertile women showed that in the endometriosis women the lower expression of PTEN mRNA was seen compared to that in the group of women with tubal infertility (*P*<0.05).

**Table 1:** The character of mRNA expression of genes, regulating apoptosis, in the endometrium of healthy women and patients with tubal infertility and endometriosis

Group	XIAP, normalized copies numbers x10^4^/µl	PTEN normalized copies numbers x10^1^/µl	HSP27 normalized copies numbers x10^4^/µl
Control group (n=13)	2.28±0.49	1.47±0.47	3.32±0.79
Tubal factor of infertility (n=73)	5.91±0.96*	2.82±0.47*	7.62±0.91*
Endometriosis (n=27)	18.69±7.22*	1.84±0.54†	8.03±2.08*

* - differences between the control group and groups of women with tubal factor and endometriosis are statistically significant (

* - *P*<0.05);

† - differences between the group of women with tubal factor and endometriosis are statistically significant (†- *P*<0.05).

### Apoptosis regulation in the endometrium of infertile women with different IVF treatment outcome

To establish the relationship between the character of endometrial apoptosis regulation and endometrial receptivity in infertile women, we have compared the endometrial apoptosis parameters in women who later entered in the IVF protocol in dependence to the outcome of IVF treatment. It was found that the presence of endometriosis is a factor, limiting the successful implantation and pregnancy outcome after IVF (Table 2). Both studied groups of infertile women were comparable to each other for the age, numbers of oocytes received and embryos transferred, but the pregnancy rate after IVF was significantly lower in infertile women with endometriosis comparing to that in infertile women with tubal factor (*P*<0.05).

**Table 2:** IVF outcome in infertile women with tubal factor and endometriosis

Study Group	Tubal factor	Endometriosis	*P* value
N^o^. of patients	53	17	
Age (years)	33.13±0.42	33.22±0.97	>0.05
N^o^. of oocytes received*	7.44±0.96	5.67±0.90	>0.05
N^o^. of embryos transferred*	1.96±0.09	1.53±0.22	>0.05
N^o^. of pregnancies (%)	47.2	17.6	<0.05
Miscarriage (%)	16.0	0.0	>0.05
Live birth (%)	39.6	17.6	>0.05

* - values are mean ± standard error

*P* - given in comparison to the tubal factor

The endometrial genes profiles were also different in women with endometriosis and tubal factor who achieved ongoing pregnancy after IVF (Table 3). In women with tubal factor of infertility the implantation success was associated with the initially high level of PTEN mRNA expression in the endometrium (*P*<0.05). In infertile women with endometriosis we didn’t find statistically significant differences in PTEN mRNA expression among endometriosis women with different IVF outcome but the ongoing pregnancy was seen in women with higher PTEN mRNA expression (*P*>0.05) (Table 3). Lower level of XIAP mRNA expression was associated with IVF treatment successes in women with endometriosis (*P*<0.05).

**Table 3:** The level of mRNA expression of genes, regulating apoptosis, in the endometrium of infertile women with different IVF treatment outcome

Parameter	Tubal factor		Endometriosis	
	Successful ongoing pregnancy (n=21)	Implantation failure (n=28)	Successful ongoing pregnancy (n=3)	Implantation failure (n=14)
XIAP, normalized copies numbers x 10^4^/µl	5.29±1.42	5.11±1.67	0.44±0.43	2.84±0.89*
PTEN, normalized copies numbers x 10^1^/µl	3.06±0.51	1.75±0.30*	5.22±3.03	1.75±0.50
HSP27, normalized copies numbers x 10^4^/µ	6.39±0.99	7.52±1.37	6.72±6.58	13.22±3.15

* - differences between the subgroup with successful ongoing pregnancy and subgroup with implantation failure are statistically significant (*- *P*<0.05)

## DISCUSSION

According to our results, the regulation of apoptosis in the endometrium of infertile women during the window of implantation is significantly impaired. We also found that the different types of infertility (tubal factor and endometriosis) were accompanied by the different changes in the expression of apoptosis-regulating genes.

In women with tubal factor infertility the high level of mRNAs expression of both anti-apoptotic (XIAP and HSP27) and pro-apoptotic (PTEN) factors was noted comparing to that in the fertile control. It is well known that XIAP is one of the important inhibitors of apoptosis, which is able to bind with caspase-9,-2 and-7 and inactivate their activity (Schimmer *et al.*, 2006). The XIAP expression is strongly elevated in patients with different types of tumors and the high level of XIAP production is associated with the poor prognosis (Schimmer *et al.*, 2006). The elevated expression of HSP27 effectively protects cells from apoptosis (Schmitt *et al.*, 2007). HSP27 has been shown to interact and inhibit components of both stress- and receptor-induced apoptotic pathways. It was demonstrated that HSP27 could prevent the activation of caspases. It does so by directly sequestering cytochrome c when released from the mitochondria into the cytosol (Schmitt *et al.*, 2007). According to the literature data during the window of implantation the increase of apoptosis in the endometrial tissue is noted (Szmidt *et al.*, 2010). It was suggested that this phenomenon regulates the establishment of endometrial receptivity and provides the adequate invasion of the implanted blastocyst in the endometrial stroma (Szmidt *et al.*, 2010). So, the high level of the production of anti-apoptotic factors might be estimated as a negative factor that can reduce the receptivity of endometrium of women with tubal factor infertility.

Surprisingly, we found that the synthesis of pro-apoptotic factor PTEN was also significantly increased in the endometrium of women with tubal factor infertility. This factor was discovered as tumor suppressor in 1997 (Zhang & Yu, 2010).

Somatic mutations of PTEN have been identified as a prevalent event in different type of tumors, particularly those of the endometrium, brain, skin and prostate (Zhang & Yu, 2010). PTEN has been shown to be a non-redundant, evolutionarily conserved dual-specific phosphatase that is capable of removing phosphates from protein and lipid substrates (Bononi *et al.*, 2011).

The primary target of PTEN is the lipid second messenger intermediate PIP3 (phosphatidylinositol 3,4,5-trisphosphate). PTEN removes the phosphate from the three-position of the inositol ring to generate PIP2 (phosphatidylinositol 4,5-bisphosphate) thereby directly antagonizing intracellular signaling through the PI3K/Akt pathway (Zhang & Yu, 2010). It is known that Akt is the major downstream effector of PI3K (phosphoinositide 3-kinase) signaling that can phosphorylate a wide array of substrates and, thus, stimulates cell growth, proliferation and survival (Zhang & Yu, 2010). Today it is well documented that PTEN function affects diverse cellular processes such as cell-cycle progression, cell proliferation, apoptosis, aging, DNA damage response, angiogenesis, muscle contractility, chemotaxix, cell polarity and stem cell maintenance (Bononi *et al.*, 2011). The requirement of PTEN for embryonic development was also established (Bononi *et al.*, 2011).

Recently the role of PTEN in the mammalian uterus as well as its requirement for proper trophoblast invasion and decidual regression was demonstrated (Laguë *et al.*, 2010). Taking into account these properties of PTEN we suggested that the high level of PTEN mRNA expression in the endometrium of patients with tubal factor infertility might be estimated as positive mechanism, which compensates the overexpression of anti-apoptotic factors and facilitates the readiness of endometrium to the implantation.

Endometrium of women with endometriosis was also characterized by the high level of XIAP and HSP27 mRNAs expression. But we didn’t find any changes in the expression of pro-apoptotic factors mRNAs in this group and the level of PTEN mRNA expression in the group of endometriosis women was significantly lower than that in infertile women with tubal factor.

Thus, the high synthesis of the anti-apoptotic factor is characteristic to the endometrium of women with endometriosis.

Earlier, it was suggested that there are some fundamental differences in the endometrium of women with endometriosis, such as resistance to apoptosis, which could contribute to the survival of the regurgitating endometrial cells into the peritoneal cavity and the development of endometriotic lesions (Harada *et al.*, 2004).

Likely, the high synthesis of XIAP and HSP27 in the endometrium can also provide the decrease of apoptosis of endometrial cells and facilitates its viability and growth in the peritoneal cavity. From another side, the high level of anti-apoptotic activity in the endometrium during the period of implantation window without counterbalanced elevated expression of the pro-apoptotic factors possibly can lead to the decrease of the endometrial receptivity of women with endometriosis.

This suggestion is confirmed both by literature data (Coccia *et al.*, 2011) and by our results about the association of endometriosis with poor IVF outcome. To elucidate the possible interaction between the character of endometrial apoptosis regulation and endometrial receptivity we have analyzed the apoptosis-associated genes expression in the endometrium of women with different outcome of IVF treatment. In women with tubal factor infertility the pregnancy achievement was associated with the maximal level of PTEN mRNA expression.

These results let us to suggest that PTEN is essential for endometrial receptivity and successful implantation.

Likely, the estimation of PTEN synthesis in the endometrium might be used as the predictor of endometrial receptivity at least in infertile women with tubal factor.

In women with endometriosis the positive IVF outcome was associated with the low level of the XIAP mRNA expression in the endometrium.

So, in this group the same association of high endometrial apoptosis during window of implantation and IVF successes was traced.

## CONCLUSION

In summary, the estimation of the activity of apoptosis in the endometrium during the window of implantation surely gives us the important information about the endometrial receptivity.

The high level of apoptosis-inducing factors synthesis in this period provides the optimal conditions for preparing of the endometrium to implantation.

The high level of PTEN mRNA expression in endometrium of infertile women with tubal factor associates with successes of IVF treatment.

The lower expression of PTEN mRNA in the endometrium of endometriosis women might be responsible for lower rate of IVF success in this group.

The further investigations of this problem would likely let us to develop new predictors of IVF outcome.
